# Supplementary material for: Design and Validation of a Brain-Controlled Hip Exoskeleton for Assisted Gait Rehabilitation Training
Source: Micromachines (Basel). 2025 Nov 29;16(12):1364. doi: 10.3390/mi16121364 (PMC12734547; doi:10.3390/mi16121364)
Supplement: Supplementary file 1 [file micromachines-16-01364-s001.zip › micromachines-4007490-supplementary.pdf]

Supporting Information for

Design and Validation of a Brain-controlled Hip Exoskeleton for Assisted Gait Rehabilitation Training

Chengjun Wang[123&], Biao Cheng[1&*], Qiang Tang[12&*], Renyuan Wu[1], Huanyu Li[1]

1*School of Artificial Intelligence, Anhui University of Science and Technology, Huainan, Anhui 232001, China.
[2]State Key Laboratory of Digital Intelligent Technology for Unmanned Coal Mining, Anhui University of Science and Technology, Huainan 232001, China.
[3]School of Artificial Intelligence, Anhui Polytechnic University, Wuhu, Anhui 241000, China.
[&]C.W., B.C. and Q.T. contributed equally to this work.
*Corresponding author: Biao Cheng. E-mail: chengbiao@aust.edu.cn. Qiang Tang. E-mail: tangqiang@whu.edu.cn.
Contributing authors: Chengjun Wang: wangchengjun@ahpu.edu.cn. Renyuan Wu: wury@aust.edu.cn. Huanyu Li: a15029238138@163.com.

This file includes:

Table S1 to S2

Table S1. Key hardware models and parameters.

Hardware name	Model	Parameters
EEG Amplifier	Brain Products actiCAP	8-channel EEG amplifier, 256/512 Hz sampling rate, 24-bit resolution, <1 μV RMS noise, USB 2.0 interface.
Electrode Cap	Brain Products actiCAP snap	Standard 10-20 system wet electrode cap with 8 parietal-occipital channels.
Main Motion Controller	Delta Tau PMAC Clipper	High-performance DSP+FPGA core, 20 Hz (50 ms) control cycle, Ethernet and CAN bus interfaces.
Low-level Logic & I/O Controller	Xilinx Zynq-7000 SoC FPGA	Real-time processor and FPGA architecture, with 1 kHz FPGA loop rate for sensor polling and PWM valve control.
Hip Joint Angle Sensor	TDK InvenSense MPU-6050	6-axis IMU (3-axis accelerometer ±8 g, 3-axis gyroscope ±1000 °/s) for hip joint angle calculation via sensor fusion.
Plantar Pressure Sensor	Interlink Electronics 402	Thin-film pressure sensor (0-100 N range) for binary gait phase detection.
Human-Robot Interaction Force Sensor	Interlink 402	Miniature piezoresistive sensor (0-78.4 N range, <0.2 mm thickness) for human-robot interface contact pressure measurement.
High-Speed Switching Valve	Festo MHJ9 Series	Piloted solenoid valve with <10 ms on/off response time for pneumatic control via FPGA PWM signals.
Pneumatic Actuation Unit	Spring-Return Single-Acting Cylinder (Generic)	Spring-return single-acting cylinder providing 1.8-3.1 N·m assistive torque.
System Pressure Sensor	SMC PSE Series	System pressure sensor with 0-1.0 MPa range for pneumatic supply monitoring.

Table S2. List of abbreviations and acronyms.

Abbreviation	Full term / Definition
BCI	Brain-Computer Interface
AR-VS	Augmented Reality and Visual Stimulation
SSVEP	Steady-State Visual Evoked Potentials
FBCCA	Filter Bank Canonical Correlation Analysis
VCR	Virtual Center of Rotation
BMI	Brain–Machine Interface
LAN	Local Area Network
KCF	Kernelized Correlation Filter
CCA	Canonical Correlation Analysis
PMAC	Programmable Multi-Axis Controller
RBF	Radial Basis Function
LR	Linear Regression
LSTM	Long Short-Term Memory
NRMSE	Normalized Root Mean Square Error
FPGA	Field-Programmable Gate Array
GND	Ground Electrode
VFD	Visual Feedback Display
SNR	Signal-to-Noise Ratio
PSD	Power Spectral Density
FEM	Finite Element Method
CIR	Correlated Instantaneous Rate
